# Evaluation of connectivity map-discovered celastrol as a radiosensitizing agent in a murine lung carcinoma model: Feasibility study of diffusion-weighted magnetic resonance imaging

**DOI:** 10.1371/journal.pone.0178204

**Published:** 2017-05-23

**Authors:** Hong Young Jun, Tae-Hoon Kim, Jin Woo Choi, Young Hwan Lee, Kang Kyoo Lee, Kwon-Ha Yoon

**Affiliations:** 1 Imaging Science Research Center, Wonkwang University Hospital, Iksan, Republic of Korea; 2 Laboratory of Pharmacogenetics, Kyung Hee University College of Pharmacy, Seoul, Republic of Korea; 3 Department of Radiology, Wonkwang University School of Medicine, Iksan, Republic of Korea; 4 Department of Radiation Oncology, Wonkwang University School of Medicine, Iksan, Republic of Korea; Northwestern University Feinberg School of Medicine, UNITED STATES

## Abstract

This study was designed to identify potential radiosensitizing (RS) agents for combined radio- and chemotherapy in a murine model of human lung carcinoma, and to evaluate the *in vivo* effect of the RS agents using diffusion-weighted magnetic resonance imaging (DW-MRI). Radioresistance-associated genes in A549 and H460 cells were isolated on the basis of their gene expression profiles. Celastrol was selected as a candidate RS by using connectivity mapping, and its efficacy in lung cancer radiotherapy was tested. Mice inoculated with A549 carcinoma cells were treated with single ionizing radiation (SIR), single celastrol (SC), or celastrol-combined ionizing radiation (CCIR). Changes in radiosensitization over time were assessed using DW-MRI before and at 3, 6, and 12 days after therapy initiation. The tumors were stained with hematoxylin and eosin at 6 and 12 days after therapy. The percentage change in the apparent diffusion coefficient (ADC) value in the CCIR group was significantly higher than that in the SC and SIR group on the 12^th^ day (Mann–Whitney U-test, p = 0.05; Kruskal–Wallis test, p < 0.05). A significant correlation (Spearman’s rho correlation coefficient of 0.713, p = 0.001) was observed between the mean percentage tumor necrotic area and the mean ADC values after therapy initiation. These results suggest that the novel radiosensitizing agent celastrol has therapeutic effects when combined with ionizing radiation (IR), thereby maximizing the therapeutic effect of radiation in non–small cell lung carcinoma. In addition, DW-MRI is a useful noninvasive tool to monitor the effects of RS agents by assessing cellularity changes and sequential therapeutic responses.

## Introduction

Lung cancer is the most common type of cancer and the leading cause of cancer-related deaths worldwide, with non-small cell lung carcinoma (NSCLC) being the main type of lung cancer. Currently, radiation and radiosensitizing (RS) chemotherapy, which trigger cancer cell death through different mechanisms, are the conventional treatment methods because concurrent RS chemotherapy and thoracic radiation resulted in increased survival rates for NSCLC patients [1–7].

More recently, drug repositioning combined with *in silico* approaches has been used to identify RS agents. Microarray technology and innovative bioinformatic frameworks, such as the Connectivity Map, are helpful web-based tools comprising a large gene expression database generated from human cancer cell lines treated with different chemicals [8,9]. The Connectivity Map is based on chemical genomics to identify drugs modulating biological processes by querying gene expression patterns [10–15]. Several studies in clinical medicine and molecular genetics have made use of this powerful tool, and Connectivity Map-based drug discovery has the potential to identify novel RS compounds.

Noninvasive imaging tools such as magnetic resonance (MR) techniques are used to evaluate the efficacy of radiation therapy as they can assess altered metabolism and normalization in treatment-responsive tumors [16,17]. Specifically, diffusion-weighted magnetic resonance imaging (DW-MRI) has been applied to detect the loss of cellularity, which is the end result of extensive necrosis [18–21], and other types of cell death such as mitotic catastrophe and apoptosis [22].

The aim of this study was to 1) identify RS agents that are potential candidates for lung cancer radiotherapy by using Connectivity Map, and 2) to evaluate their *in vivo* effect using DW-MRI in conjunction with radio- and chemo-combination therapy in a xenograft lung cancer murine model.

## Materials and methods

### *In silico* study

NSCLC samples were characterized on the basis of their sensitivity or resistance to IR-induced apoptosis as previously described [23,24]. Gene expression data were normalized using dChip [25] and were filtered with a max-min = 100 and max/min = 4. The probe sets that correlated with the sensitive/resistant distinction were determined using signal-to-noise statistics and permutation testing. The 157 probe sets with p < 0.0005 were part of an IR sensitivity/resistance profile and were used for subsequent comparisons.

The Connectivity Map included instances that represented a radiation treatment and control pair, and the list of genes was ordered according to the extent of the differential genome-wide expression of the genes between the radiation treatment and control pair. The connectivity score consisted of a group of perturbagens with the enrichment scores from the up- and downregulated genes. Instances were rank-ordered in descending order of the connectivity score. All instances in the database were then ranked according to their connectivity scores; those at the top were most strongly correlated to the query signature and those at the bottom were most strongly anti-correlated [26]. All data used for the connectivity map were found at http://www.broad.mit.edu/cmap/ and in the Gene Expression Omnibus. The RS candidate agents were nominated on the basis of their connectivity score-based rank. Finally, we selected those agents that showed concurrent results in both A549 and H460 cells.

### Radiation and chemotherapy reagents

A549 and H460 human lung carcinoma cells were purchased from the Korean Cell Line Bank, and were cultured in Roswell Park Memorial Institute (RPMI) 1640 medium supplemented with 10% fetal bovine serum (FBS) and 1% penicillin–streptomycin. The cells were cultured at 37°C in a humidified environment containing 7.5% CO_2_. Celastrol and dimethyloxalylglycine (DMOG) were purchased from Sigma-Aldrich Corp. (St. Louis, MO, USA). For the *in vitro* treatments, celastrol was dissolved in dimethyl sulfoxide (DMSO; Sigma-Aldrich Corp.) to a concentration of 0.4 mM. DMSO in the final solution did not exceed 0.2% (v/v). DMOG was dissolved in phosphate-buffered saline to a concentration of 25 mM and was further diluted to the appropriate final concentration in RPMI 1640 with 10% FBS.

To compare the *in vivo* RS efficacy of celastrol in the A549 carcinoma murine model, the mice were randomly divided into three groups of six each: a single ionizing radiation (SIR) group, single celastrol (SC) group, and celastrol-combined IR (CCIR) group. The SC and CCIR group were treated with celastrol (2 mg/kg daily for 5 days), and the SIR group was treated with vehicle (10% DMSO, 70% kolliphor EL/ethanol [3:1] and 20% PBS). Celastrol or vehicle solution was injected intraperitoneally. The posterior base of the mouse tumor was aligned with the treatment isocenter using the On-board Imager system (Varian Medical Systems, Palo Alto, CA, USA). A single IR dose (10 Gy) was applied using a 6-MV photon beam with a nominal dose rate of 6 Gy/min at the isocenter. Each mouse was protected with a lead cover for reducing normal tissue injury with only tumor exposed, allowing local irradiation.

### Cell viability assay

A549 and H460 cells were seeded into 96-well plates and were pretreated with celastrol (0.125–4 μM) or DMOG (15.625–250 μM) at increasing concentrations for 4 h. Cell viability was determined using a water-soluble tetrazolium salt (WST-1) cell viability assay per the manufacturer’s instructions (Premix WST-1, Takara, Japan). The absorbance was measured at 450 nm using a Bio-Rad plate reader (Bio-Rad Model 680 system with Microplate Manger 5.2 software; Bio-Rad, Hercules, CA, USA). The values were calculated as the ratio of celastrol-treated cells to baseline control cells, and were the average from four independent experiments.

### Colony formation assay

A549 and H460 cells were seeded into 60-mm dishes at 500 cells per dish. Celastrol (2 μM) and DMOG (250 μM) were added to each dish 4 h prior to IR treatment (2–10 Gy). After 15 days, the media were removed, the cells were stained with 1% crystal violet (Sigma-Aldrich Corp., St. Louis, MO, USA) in 10% ethanol, and the number of cells was counted. The experiments were performed in triplicate. The highest ranking drug for the *in vivo* validation in the A549 xenograft murine model was selected on the basis of the results of the colony formation assay.

### Lung tumor xenograft mouse model

Experimental procedures were performed with approval from the Institutional Animal Care and Use Committee of Wonkwang University (approval No. WKU14-93). Nude mice were purchased from Central Laboratory Animal, Inc., (Seoul, Korea) and were bred in-house in our pathogen-free animal facility. A total of 18 male nude mice (aged 5 weeks and weighing 20 g each) were used for this study. All the mice used in this study were maintained in an individual ventilation cage under specific pathogen-free condition in a 12-h light-dark cycle and were provided standard mouse chow ad libitum. The body weight of all mice was measured daily during the experiment. All procedures adhered to the ARRIVE Guidelines for reporting animal research [27]. A checklist is included in S1 Checklist.

A549 cells were removed from the culture flasks using trypsin. About 5 × 10^7^ cells in 100 μL of media were immediately injected in the back subcutaneously of each mouse. The assessment of tumor radiosensitivity modification by the selected agents was initiated when the tumor volume was about 150–200 mm^3^. The tumor volume was measured more precisely using T2-weighted MRI (fast spin-echo). The tumor volumes were calculated from the entire region of interest (ROI) drawn around the tumor and by the perimeter method using the formula: volume = slice thickness X (A_1_+A_2_+…A_n_), where A_n_ is the area of the n^th^ slice of the tumor [28].

### Diffusion-weighted magnetic resonance imaging

Approximately 4–6 weeks after inoculation of the mice with the A549 cells, volume-matched tumors were imaged before treatment and 3, 6, and 12 days following treatment with SIR, SC, or CCIR. The mice were placed in an induction chamber filled with 4% isoflurane in oxygen to induce anesthesia. During imaging, the mice remained anesthetized using 1.5% isoflurane in oxygen. The mice recovered from the anesthesia between the images acquisitions.

During MRI acquisition, the animals were anesthetized using 1.5% isoflurane in oxygen-enriched air supplied with a facemask. The respiration rate and rectal temperature were monitored using a small-animal monitoring system (SA Instruments, Stony Brook, NY, USA). MRI acquisitions were performed using a 4.7-T horizontal MRI device (BioSpec; Bruker, Ettlingen, Germany) with a 65-mm diameter shielded gradient. The experiments were performed using a 38-mm internal diameter birdcage coil. A diffusion-weighted spin echo sequence was used with the following acquisition parameters: echo time = 32 ms; repetition time = 6600 ms; b-values = 0, 100, 200, 300, 400, 600, and 800 sec/mm^2^; slice thickness = 1 mm; 22 slices; field-of-view = 4 × 4 cm; and 6 averages. Images were acquired in a 128 × 128 matrix resulting in a resolution of 0.0312 cm/pixel. ADC maps were constructed from diffusion-weighted imaging sequences with b-values 200–800 sec/mm^2^ using the ParaVision 4.0 software (Bruker BioSpin, Ettlingen, Germany). The diffusion coefficient for the entire tumor ROI, as well as the ADC map, was calculated using the image-sequence analysis tool.

### Histological analysis

Six and twelve days after treatment initiation, the three mice in each group were euthanized by cervical dislocation for histological analysis of the subcutaneous tumors. No mice died before the euthanasia process in all groups. The tumors were fixed in 10% formalin and were subsequently paraffin-embedded before staining with hematoxylin and eosin (H&E). The percentage of necrosis was determined by measuring the total dimension of the field-of-view and comparing it with the dimension of the necrotic area.

### Statistical analysis

The non-parametric Kruskal–Wallis (KW) test was used for comparing the mean between the different treatment groups. The Mann–Whitney U (MWU) test was used as a post hoc test if significant differences were found. The MWU was used for comparisons between two independent groups. The Spearman’s rank-order correlation test was used to investigate correlations between quantitative variables. The data are represented as the mean ± SEM, unless otherwise stated. Statistical analysis was performed using the SPSS software version 11.5 (SPSS, Inc., Chicago, IL, USA). Statistical significance was set at p ≤ 0.05.

## Results

### Analysis of RS candidates using a connectivity map

**Fig 1:**
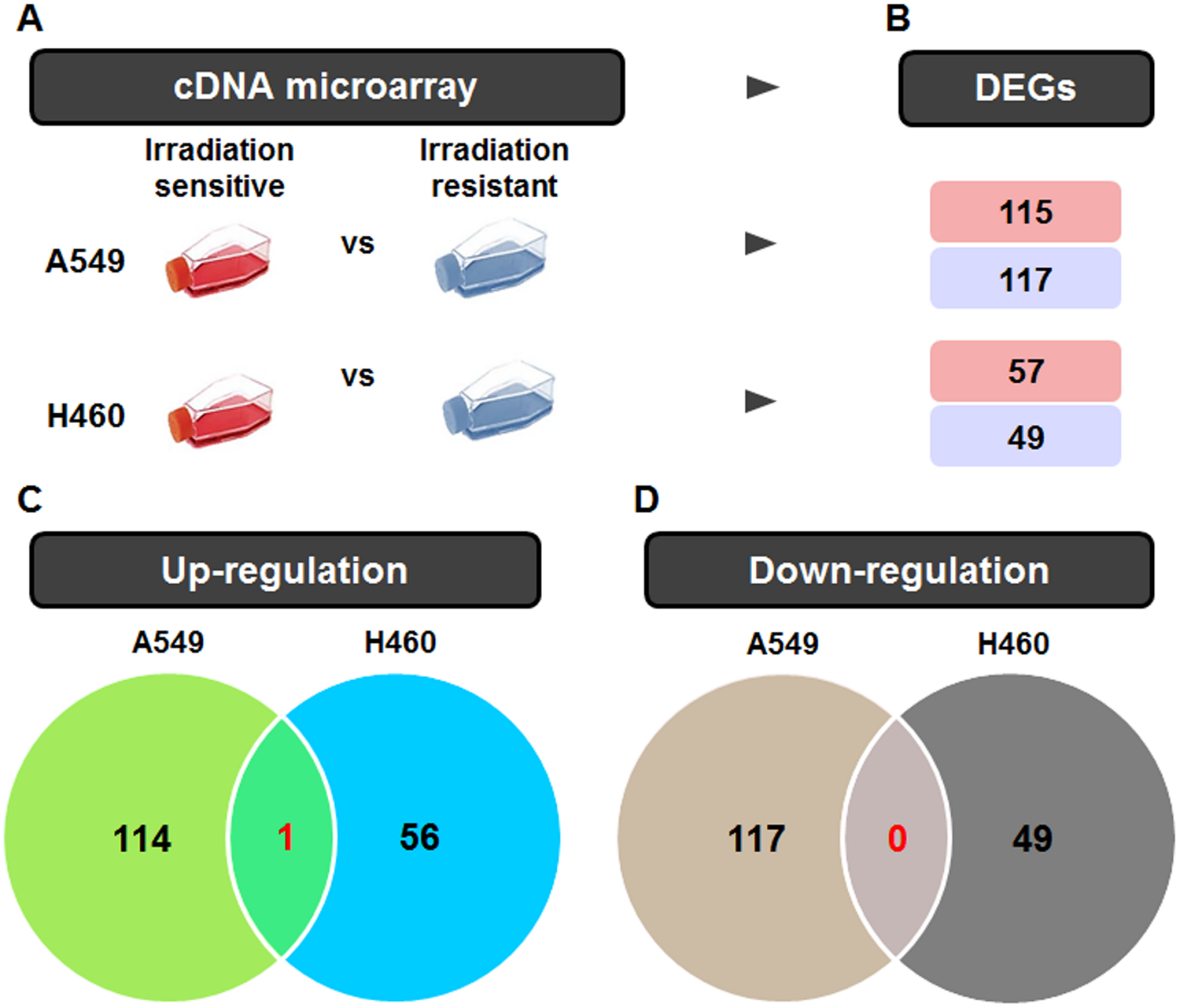
Venn diagram summarizing the differentially expressed genes (DEGs) in radiation-treated A549 and H460 cells. (A) The radiation-related gene expression signatures of A549 and H460 cells were collected using cDNA microarrays. (B) The differential gene expression in each group was shown for the unique upregulated and unique downregulated DEGs. (C) The number of up-regulated genes in each group and the number shared by the different groups are depicted in a Venn diagram. (D) The number of down-regulated genes in each group is also expressed in a Venn diagram.

The gene expression data of the A549 and H460 cells after IR were collected and reanalyzed using statistical comparisons with thresholds of p < 0.05 and a 1.5-fold difference. Among the genes analyzed, 115 (up) and 117 (down) genes were regulated in A549 cells, and 57 (up) and 49 (down) genes were regulated in H460 cells, with 1 (up) and 0 (down) overlapping genes (Fig 1).

**Fig 2:**
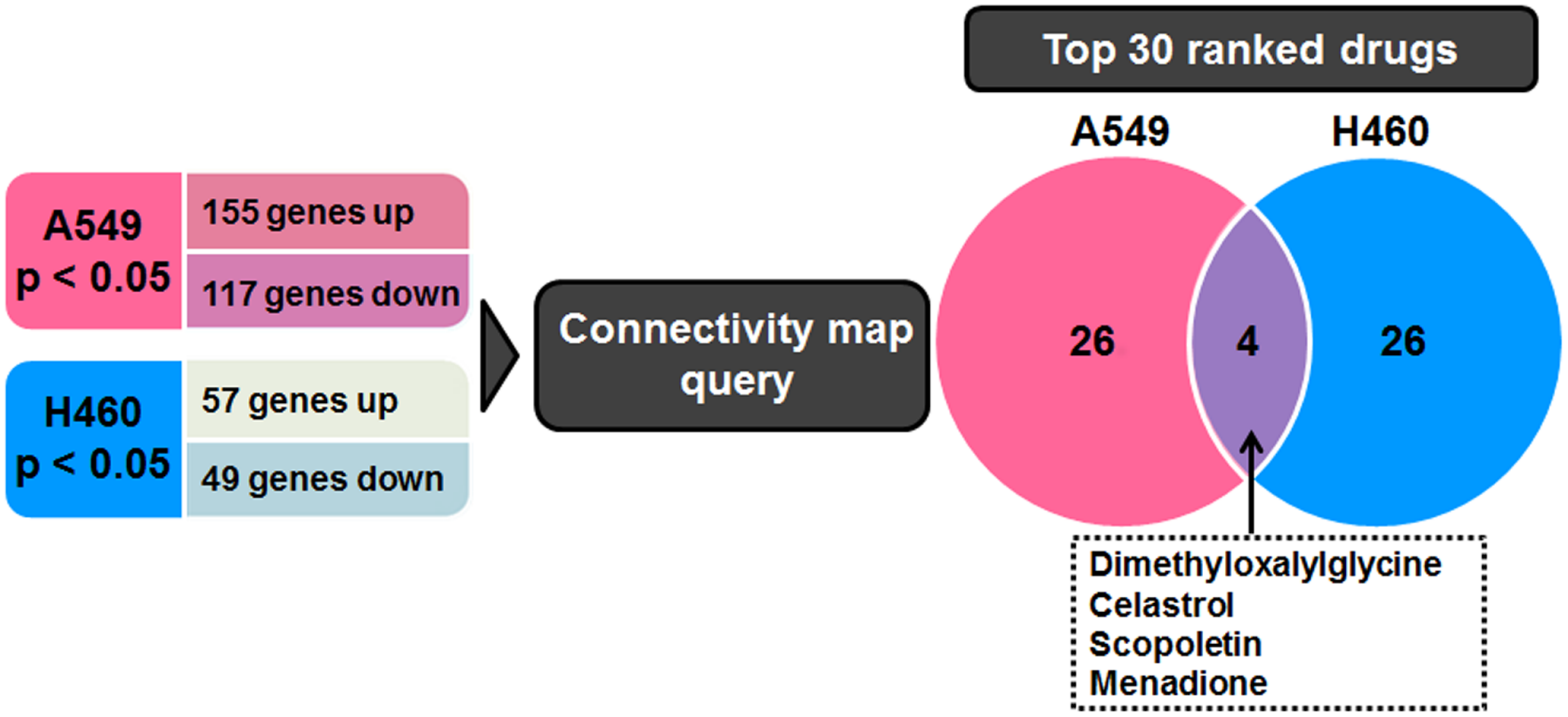
Identification of candidate radiosensitizers using a connectivity map. Genes with a false discovery rate-adjusted p value (p < 0.05) and a fold change >1.5 were considered DEGs. Connectivity mapping between A549 and H460 cells gene signatures is shown. Four drugs show overlap in the top 30 drug list for both lung cancer cell lines.

Based on the overlapping signature genes, we independently listed the top 30 drugs for A549 and H460 cells (Table 1), in addition to the drugs that showed significant effects in the meta-analysis (FDR, p < 0.05). The drugs present in both drug lists are DMOG, celastrol, scopoletin, and menadione (Fig 2). Among these, celastrol and DMOG have the highest ranks.

**Table 1:** The top 30 drugs for A549 and H460 cells that were selected using connectivity map.

Rank	A549	Rank	H460
**1**	Fisetin	**1**	U0125
**2**	DMOG^a^	**2**	DMOG^a^
**3**	Celastrol	**3**	Pancuronium bromide
**4**	N-phenylanthranilic acid	**4**	Celastrol
**5**	Blebbistatin	**5**	Budesonide
**6**	Etomidate	**6**	Tioguanine
**7**	Tyrphostin AG-1478	**7**	Gefitinib
**8**	Chrysin	**8**	Oxamic acid
**9**	Quinostatin	**9**	Bromperidol
**10**	Tyrphostin AG-825	**10**	Dopamine
**11**	Mevalolactone	**11**	Isoconazole
**12**	Demecolcine	**12**	2-Deoxy-D-glucose
**13**	Menadione	**13**	Ifenprodil
**14**	5109870	**14**	Ouabain
**15**	Phenyl biguanide	**15**	BAS-012416453
**16**	Fluorometholone	**16**	Fenoterol
**17**	Scopoletin	**17**	Salsolinol
**18**	Ursodeoxycholic acid	**18**	PHA-00816795
**19**	Thapsigargin	**19**	3-Aminobenzamide
**20**	Etanidazole	**20**	Levcycloserine
**21**	Urapidil	**21**	Vinpocetine
**22**	Phenindione	**22**	Scopoletin
**23**	Oxybutynin	**23**	Digoxin
**24**	Piperlongumine	**24**	Cantharidin
**25**	Moxonidine	**25**	Dexverapamil
**26**	Atropine methonitrate	**26**	Difenidol
**27**	Remoxipride	**27**	Omeprazole
**28**	Ivermectin	**28**	Sanguinarine
**29**	Cantharidin	**29**	Menadione
**30**	Artemisinin	**30**	Rimexolone

^a^Dimethyloxalylglycine

### The effect of combined treatment on cell survival and proliferation

Cell viability was evaluated using the WST-1 assay (S1 Fig). The viability of celastrol-treated (4 μM) A549 and H460 cells was significantly decreased compared to that of untreated cells (p < 0.01). The maximal non-toxicity dose of celastrol was 2 μM in A549 and H460 cells as confirmed using the WST-1 assay. The viability of DMOG-treated (250 μM) H460 cells was significantly increased compared to that of untreated cells (p < 0.01). We found that the maximal non-toxicity dose of DMOG was 250 μM, and was higher in A549 and H460 cells according to the WST-1 assay.

**Fig 3:**
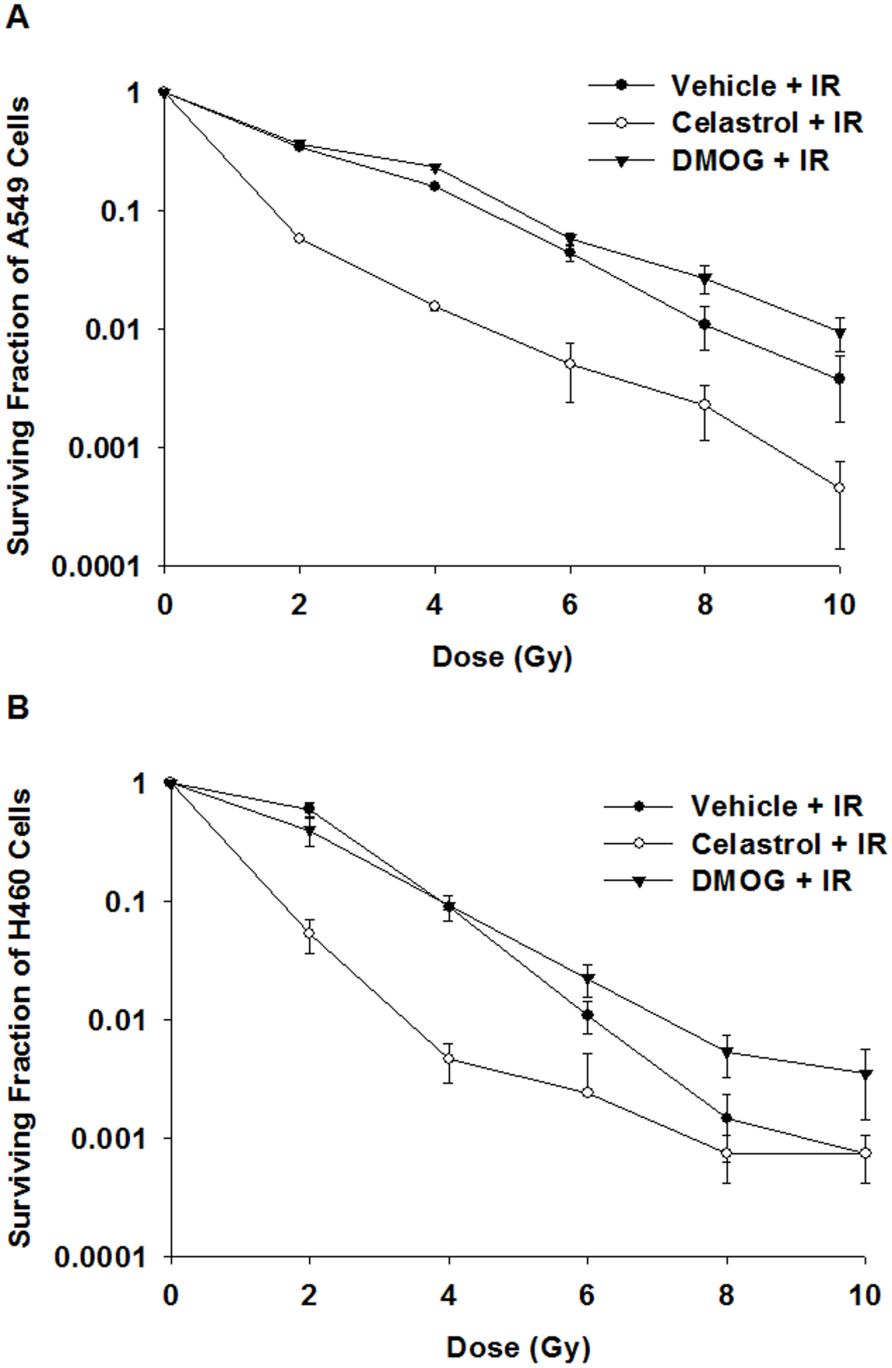
The effect of celastrol-combined IR treatment on cell survival and proliferation. The clonogenic survival was significantly decreased in both (A) A549 and (B) H460 cells exposed to an IR of 2–6 Gy and celastrol treatment (p ≤ 0.05). DMOG did not significantly reduce the survival of A549 and H460 cells exposed to these IR doses (p > 0.05). IR, ionizing radiation; DMOG, dimethyloxalylglycine.

A clonogenic assay showed that celastrol at 2 μM significantly decreased the survival of A549 cells exposed to an IR of 2–10 Gy, and of H460 cells exposed to an IR of 2–6 Gy compared to that of celastrol-untreated cells (p ≤ 0.05) (Fig 3A). On the contrary, DMOG at 250 μM significantly increased the survival of both A549 and H460 cells exposed to an IR of 8–10 Gy compared to that of DMOG-untreated cells (p ≤ 0.05) (Fig 3B). Celastrol was selected among the candidate drugs for *in vivo* validation in a A549-xenograft murine model on the basis of these results.

### *In vivo* tumor growth measurement

To determine if celastrol reduced tumor growth, the tumor volumes in the mice were measured using T2-weighted MR imaging. A549 tumor growth was decreased irrespective of celastrol or IR treatment; however, we observed significant differences at days 3 and 6 between the SIR with vehicle, SC, and CCIR group (S2A Fig). Notably, the CCIR group showed significantly suppressed tumor growth at day 12 (p < 0.05, MWU). There was no significant difference in the reduction of body weight between all 3 groups (S2B Fig), suggesting low toxicity.

### Diffusion-weighted magnetic resonance imaging analysis

**Fig 4:**
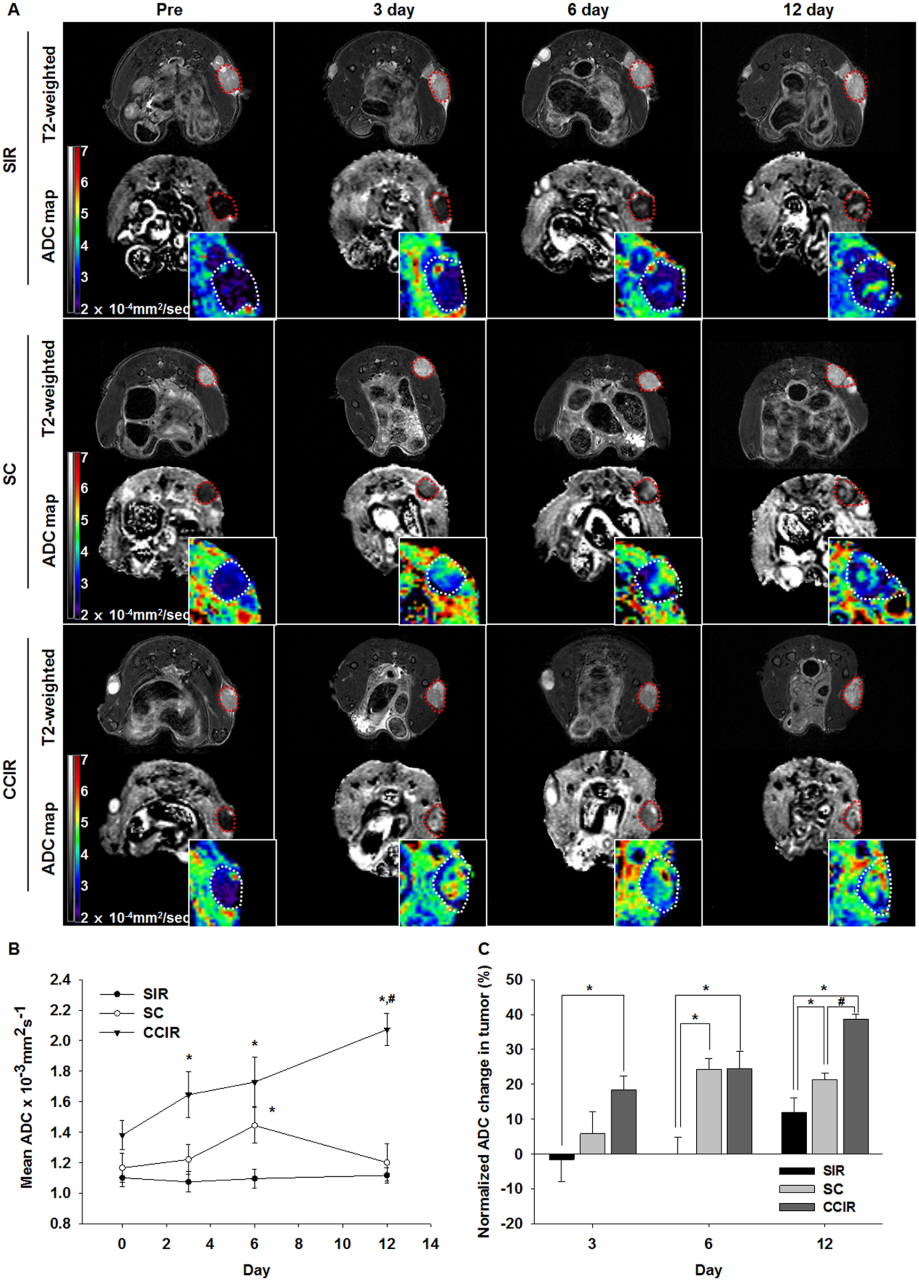
Diffusion-weighted image analysis of the tumor response to treatment. (A) T2-weighted images and apparent diffusion coefficient (ADC) maps obtained before, during, and at different times after therapy in mice treated with ionizing radiation (IR, 10 Gy), celastrol (2 mg/kg/5 days), or IR combined with celastrol therapy. The tumors were indicated with the dotted contours. The intratumoral ADC value (B) and ADC change (C) measured at 3, 6, and 12 days after the mice had been treated with IR (10 Gy), celastrol (2 mg/kg/5 day), or the combination therapy. The data represent the mean ± SEM. Statistically significant differences between the groups are indicated by the following symbols: *p ≤ 0.05 vs SIR; ^#^p ≤ 0.05 vs SC. SIR, single ionizing radiation; SC, single celastrol; CCIR, celastrol-combined ionizing radiation.

DW-MRI was used to monitor the response of the A549 tumor xenografts to the celastrol and/or IR treatments. Representative images from tumors treated with SIR, SC, or CCIR are shown in Fig 4A. The ADC values of the tumors were calculated (mean ± SEM) (all voxels) for each group at 0, 3, 6, and 12 days after treatment (Fig 4B). The ADC changes for the four groups on Day 0 were 0.001216 ± 0.000055 mm^2^/s on average (n = 18). The mean ADC increases in the CCIR group on the 12^th^ day were significantly higher than those in the SC and SIR groups (MWU, p = 0.05). The mean ADC changes of the CCIR, SC, and SIR groups were 38.66 ± 1.55%, 21.34 ± 1.79%, and 11.89 ± 4.21%, respectively, 12 days after treatment initiation (Fig 4C). The ADC change in the CCIR group was significantly higher than that in the SIR group on day 3 (MWU, p < 0.05) and in the SIR group on day 6 (MWU, p < 0.05; and KW, p < 0.005) after treatment initiation.

### Histological analysis

**Fig 5:**
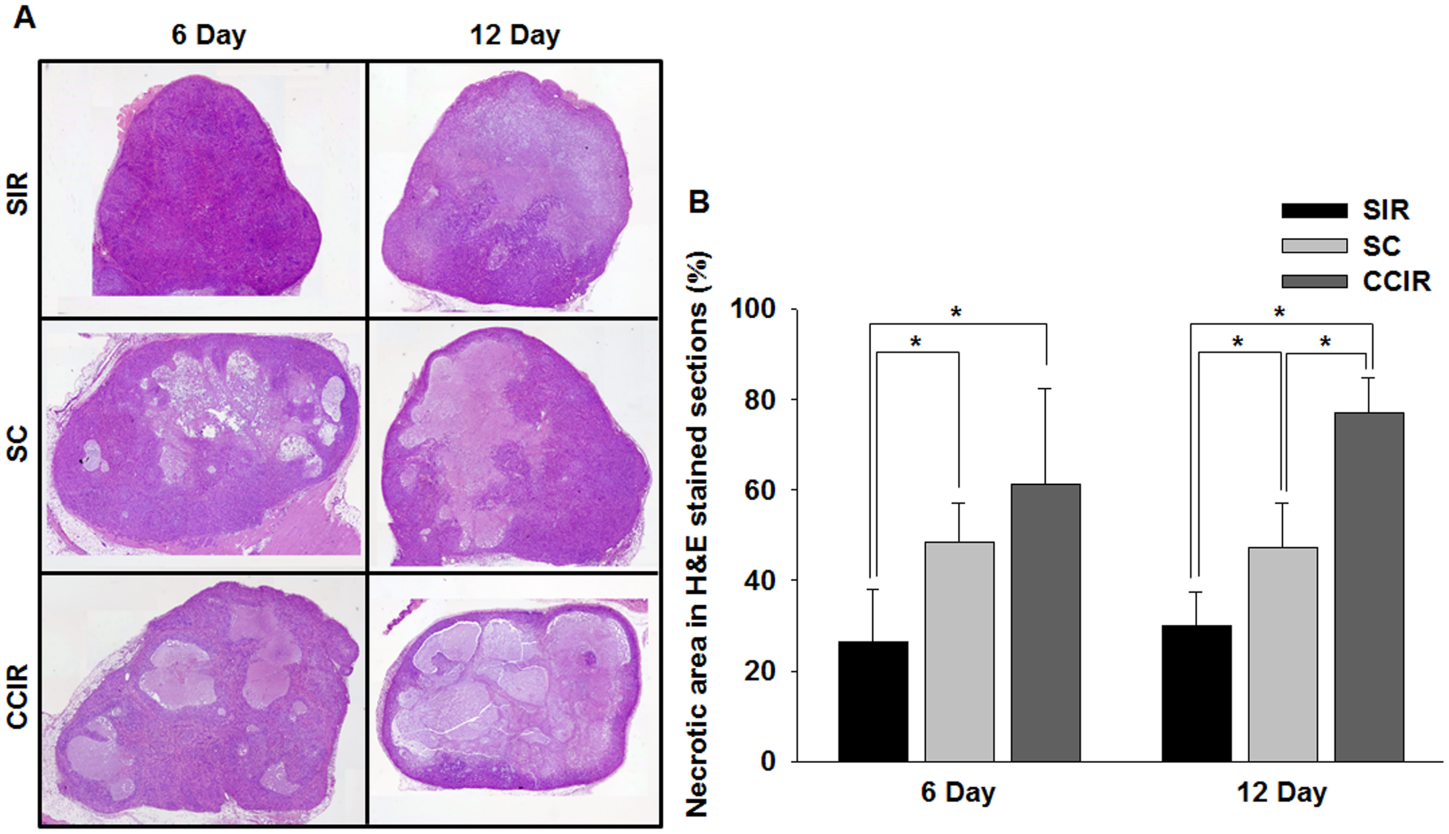
Histologic analysis of the tumor response. (A) The mean percentage tumor necrotic fraction was determined from hematoxylin and eosin- (H&E) stained sections after ionizing radiation (IR), celastrol, and combined IR celastrol therapy at day 6 and 12 after treatment initiation (original magnification, ×100). (B) Graph of the percentage necrotic area in H&E-stained sections. Tumors treated with the combined IR and celastrol therapy showed a significantly (p = 0.05) larger necrotic area at day 6 and 12 than that observed in the IR and celastrol mono-treatment groups. *p = 0.05 (statistically significant). SIR, single ionizing radiation; SC, single celastrol; CCIR, celastrol-combined ionizing radiation.

**Fig 6:**
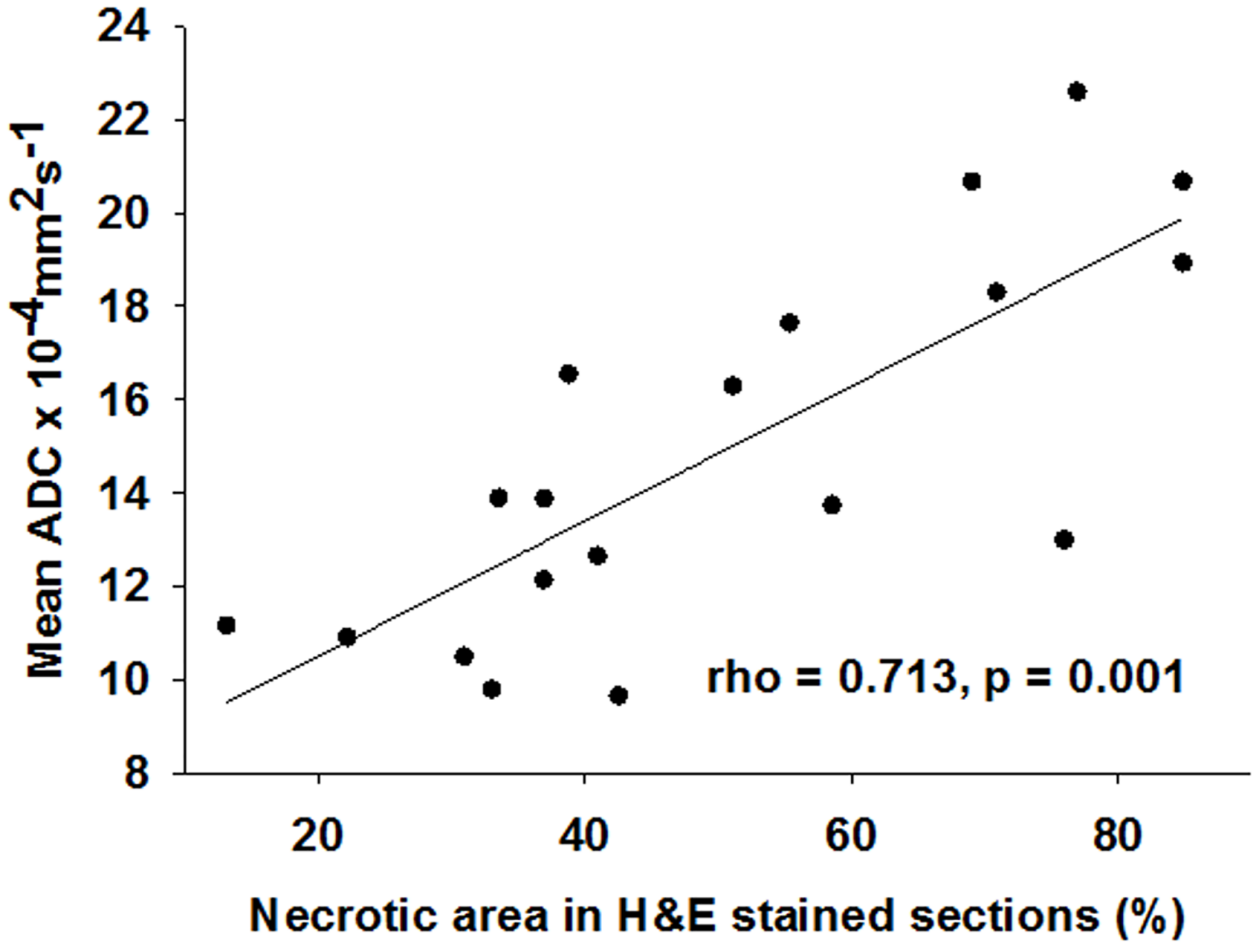
Correlation between the mean ADC change and percentage tumor necrotic fraction. The mean ADC values correlated with the percentage tumor necrotic fraction calculated from the H&E-stained sections from the A549 tumors. Spearman’s rank-order correlation test was applied to calculate the Spearman correlation coefficient (rho = 0. 713, p = 0.001). The solid lines are the result of the linear regression on the data.

Histological studies of tumor sections were performed *post mortem* to visualize the necrotic tissue in the experimental and control mice. Fig 5A shows representative micrographs of H&E- and TUNEL-stained tumor sections at 6 and 12 days after the initial treatment, respectively. On day 6, the tumor necrosis fraction calculated from the H&E-stained tumor sections was 26.53 ± 11.63%, 48.38 ± 8.61%, and 61.23 ± 21.21% in the SIR, SC, and CCIR group, respectively (Fig 5B). Notably, the necrotic fraction on day 12 was significantly increased in the CCIR group (76.92 ± 7.91%) compared to that in the SIR (29.99 ± 7.41%) and SC group (47.32 ± 9.74%) (MWU, p = 0.05; and KW, p = 0.05). As shown in Fig 6, a significant correlation (Spearman’s rho correlation coefficient of 0. 713, p = 0.001) existed between the mean percentage tumor necrotic area and the mean ADC increase measured after therapy initiation. These data suggested that there was an association between the mean ADC values and the increase in apoptosis and necrosis.

## Discussion

In this study, we analyzed the genomic signature of human lung cancer cells after radiation to identify potential RS agents for combined radio- and chemotherapy of lung cancer. RS agents were subsequently identified using Connectivity Map analysis of the resultant genomic signature. We also used DW-MRI in an *in vivo* murine model of human lung cancer to determine if the combined therapy consisting of RS agents and radiation was effective. To this end, we assessed the cellularity changes and sequential therapeutic responses and compared these to the same parameters in radiation-only controls. We found that the efficacy of IR combined with Connectivity Map-discovered RS agents might be determined with noninvasive DW-MRI to detect tumor therapeutic responses.

The Connectivity Map integrated our data to identify stable lists of candidate therapeutics by automated drug repurposing. It identified the following four RS candidates: DMOG, celastrol, scopoletin, and menadione. Of these, celastrol is a pentacyclic triterpene extracted from the plant, *Tripterygium wilfordii* Hook F (thunder god vine), and is used as a natural medicine in China for many years [29]. As shown in recent studies, celastrol has diverse cellular effects such as angiogenesis suppression, antioxidation, and anticancer activity [30–33]. Our present study demonstrates that the Connectivity Map is a very powerful tool to search for candidate RS agents. For example, it revealed the potent anticancer activity of celastrol in IR-induced tumor cells as shown using clonogenic survival assays (Fig 3).

DW-MRI is based on the mobility of water molecules within tissue *in vivo*. Water movement is less restricted in necrotic areas compared to that in viable tissue because of the decrease in cellularity in necrotic regions and a concomitant increase in the extracellular space. DW-MRI is used to discriminate between healthy and malignant tissue, and to assess tumor responses to chemotherapy, IR, and gene therapy [34–38]. In this study, DW-MRI detected the tumor response sequentially 0–12 days after initiation of the IR and celastrol therapy in a preclinical lung tumor model. Further, we showed that the therapeutic efficacy of celastrol in combination with IR could be assessed by measuring the percentage change in the ADC values. One of the interesting findings of our study was the significant decrease in the ADC values in the SIR group at day 3 after therapy initiation, while the ADC values were increased in the SC and CCIR group at this same time point. It was presumably caused by the increased tumor cell density over time leading to a decrease in extracellular water in the SIR group. The increase in the ADC values in the SC and CCIR group on 6 day after therapy initiation was likely caused by an increase in celastrol-induced apoptosis during the 5-day infusion period. At day 12 after therapy initiation, the percentage change in the ADC values indicated that the therapeutic efficacy in the CCIR group was significantly higher than that in all other groups. This result suggested that the IR-celastrol combination therapy might have significantly increased the number of apoptotic cells as shown by DW-MRI, which highly correlated with the histological measurements of necrotic areas in the H&E analysis. This trend of restricted diffusion was likely due to edema induced by acute ischemia in the tumor cells and secondary water influx into the intracellular space, with a relative decrease in the extracellular space.

We acknowledge that our study has some limitations. First, our study was limited by the utilization of only one animal tumor model. Thus, further investigation is needed to determine if the interaction between RS agents and IR can be generalized to other tumor types. Second, the mechanism of action underlying the radiosensitizing parameters of celastrol has not been investigated in this study. Some studies using cancer cells have reported that the efficacy of celastrol as an RS agent is similar to that of a heat shock protein 90 inhibitor and a p53 activator in combination with radiation therapy [33,39]. Additional studies are needed to clarify the *in vivo* biological processes affected by candidate RS agents using specific molecular imaging in animal tumor models.

In summary, we found that celastrol, a novel RS agent, increased the therapeutic effect of radiation in a lung cancer mouse model. Further, we showed that DW-MRI might be a useful noninvasive tool to monitor responses to RS agents as evidenced by cellularity changes and sequential therapeutic responses.

## Supporting information

S1 Checklist Completed ‘‘The ARRIVE Guidelines Checklist” for reporting animal data in this manuscript. (PDF)

S1 Data sheet The individual data points from which the figures were derived. (XLSX)

S1 Fig Cell viability after treatment with the candidate radiosensitizing agents. A549 and H460 cells treated with increasing concentrations of celastrol (A, B) or DMOG (C, D) for 4 h; cell viability was determined with a water-soluble tetrazolium salt (WST-1) reagent. The results are expressed as the percentage cell viability (% of untreated cells). The data represent the mean ± standard deviation. **p < 0.01 (statistically significant). DMOG, dimethyloxalylglycine.
(TIF)

S2 Fig Analysis of the tumor response to treatment with the candidate radiosensitizing agents. Graphical representation of the percentage change in tumor volume (A) and weight (B) in mice treated with ionizing radiation (IR), celastrol, or a combination of IR and celastrol. The data represent the mean ± standard deviation. *p < 0.05 (statistically significant). SIR, single ionizing radiation; SC, single celastrol; CCIR, celastrol-combined ionizing radiation.
(TIF)
