# Supplementary figures and images for: Evaluation of connectivity map-discovered celastrol as a radiosensitizing agent in a murine lung carcinoma model: Feasibility study of diffusion-weighted magnetic resonance imaging
Source: PLoS One. 2017 May 23;12(5):e0178204. doi: 10.1371/journal.pone.0178204 (PMC5441657; doi:10.1371/journal.pone.0178204)

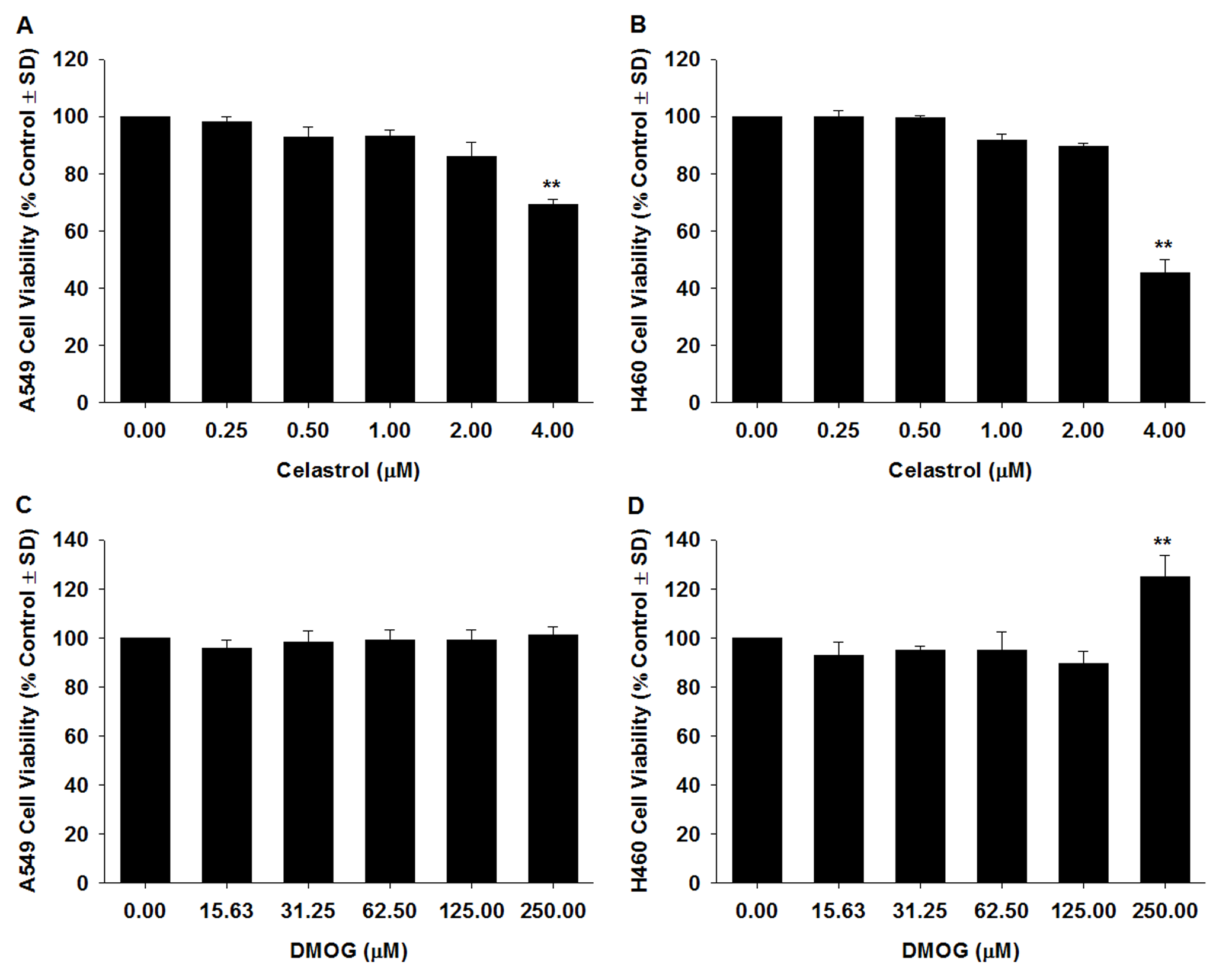

Supplement: S1 Fig — A549 and H460 cells treated with increasing concentrations of celastrol (A, B) or DMOG (C, D) for 4 h; cell viability was determined with a water-soluble tetrazolium salt (WST-1) reagent. The results are expressed as the percentage cell viability (% of untreated cells). The data represent the mean ± standard deviation. **p < 0.01 (statistically significant). DMOG, dimethyloxalylglycine. (TIF) [file pone.0178204.s003.tif]

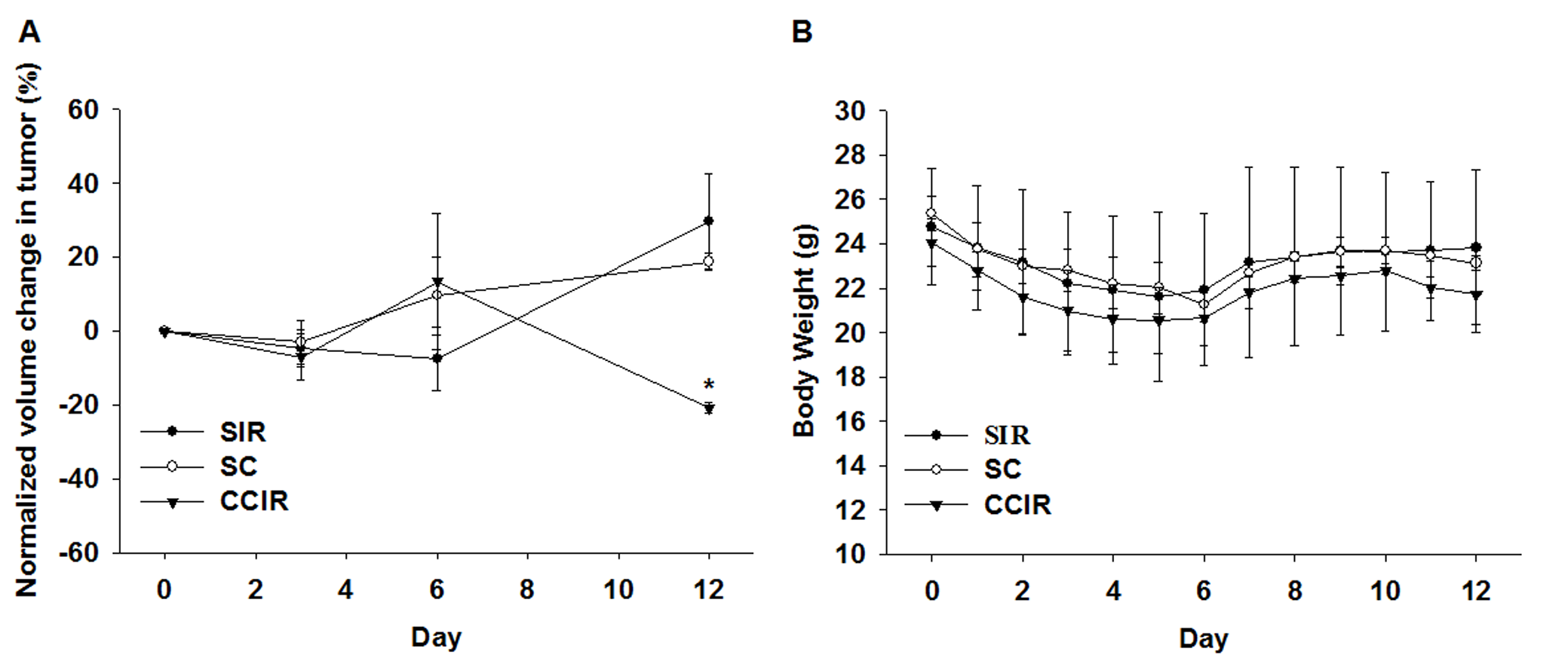

Supplement: S2 Fig — Graphical representation of the percentage change in tumor volume (A) and weight (B) in mice treated with ionizing radiation (IR), celastrol, or a combination of IR and celastrol. The data represent the mean ± standard deviation. *p < 0.05 (statistically significant). SIR, single ionizing radiation; SC, single celastrol; CCIR, celastrol-combined ionizing radiation. (TIF) [file pone.0178204.s004.tif]
